# The clinical utility of exome sequencing for risk stratification in celiac disease

**DOI:** 10.70962/jhi.20260051

**Published:** 2026-09-01

**Authors:** Talha Asif, Michele Akalay, Wendy K. Chung, Peter H.R. Green, Xiao-Fei Kong

**Affiliations:** 1 Division of Digestive and Liver Diseases, Department of Internal Medicine, University of Texas Southwestern Medical Center, Dallas, TX, USA; 2 Division of Pediatric Gastroenterology, Hepatology, and Nutrition, Department of Pediatrics, University of Texas Southwestern Medical Center, Dallas, TX, USA; 3 Celiac Disease Center, Columbia University, New York, NY, USA; 4 Department of Pediatrics, Harvard Medical School, Boston Children’s Hospital, Boston, MA, USA; 5 McDermott Center for Human Growth and Development, University of Texas Southwestern Medical Center, Dallas, TX, USA

## Abstract

The potential utility of genetic testing among individuals with a positive celiac disease (CeD) family history remains limited. We performed whole-exome sequencing in a cohort of 107 cases with CeD and 117 unaffected relatives from 66 families. We assessed fourteen HLA-DQ genotypes, based on combinations of four risk haplotypes. Our cohort was predominantly of European ancestry (87%). Compared with at-risk controls, CeD cases showed a significant enrichment of high-risk (22.4 vs. 10.3%) and moderate-risk (64.4 vs. 35.9%) HLA-DQ genotypes. Stratification based on the weighted impact of fourteen HLA-DQ genotypes yields a more accurate risk assessment than assuming equal contributions from four risk haplotypes. The HLA-B*08:01 allele was more frequent in CeD patients than in controls (32.2 vs. 16.7%). Among individuals of European ancestry, the AH8.1 long haplotype was present in 62% of cases vs. 49% of controls. Whole-exome sequencing enables stratification of first-degree relatives into discrete risk categories, identifying 10% as high risk and ∼50% as having negligible genetic risk.

## Teaser

First-degree relatives of patients with celiac disease have an estimated ∼10% lifetime risk of developing the condition, and periodic screening is therefore recommended for at-risk family members. Asif et al. from UTSW report that only ∼10% of first-degree relatives carry high genetic risk, while approximately half fall into a low genetic risk category.

## Graphical Abstract

**Figure:**
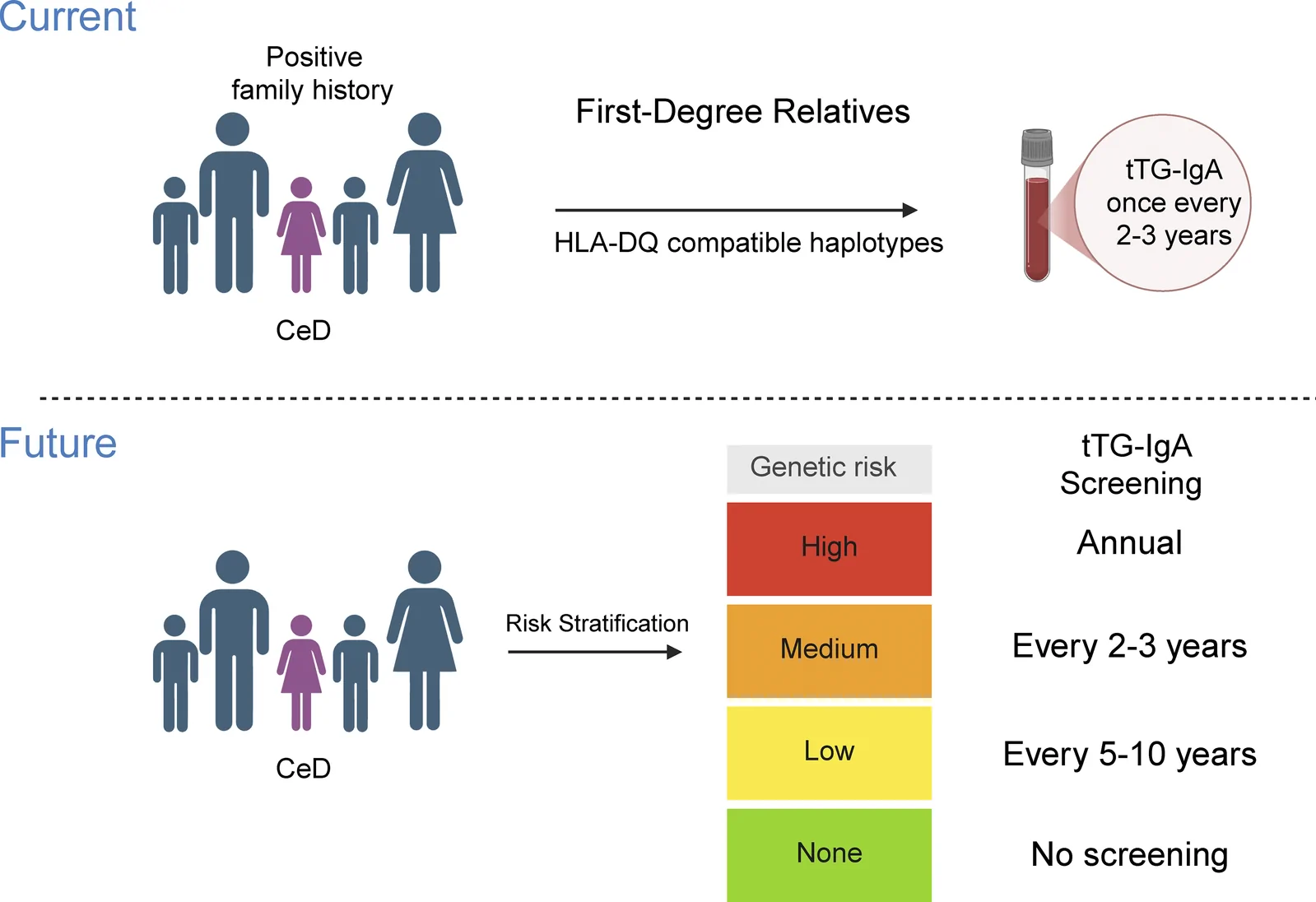

## Introduction

Celiac disease (CeD) is an immune-mediated gastrointestinal disorder precipitated by dietary gluten, with an estimated global prevalence of ∼0.7–1.4% (1, 2, 3). Prevalence is substantially higher among first-degree relatives (7–11%) (4, 5). However, studies report 43–75% of CeD cases remain undiagnosed (2, 3). Untreated CeD can lead to persistent gastrointestinal inflammation and is associated with long-term morbidities, including nutritional deficiencies, osteoporosis, anemia, and increased risk for intestinal cancer (6, 7). HLA-DQ status is historically classified by serotypes based on specific antibodies (e.g., DQ2 and DQ8) that recognize the HLA-DQ heterodimer, a protein complex formed by the pairing of a *DQA1*-encoded α chain and a *DQB1*-encoded β (8). Current HLA testing directly genotypes the *DQA1* and *DQB1*, either using allele-specific PCR or next-generation sequencing (9, 10). Both DQB1*02:02 and DQB1*02:01 alleles can generate a DQ heterodimer that is recognized by the DQ2-specific antibody, as the antibody primarily targets the β chain. However, their associated risks for CeD differ due to distinct linkage disequilibrium patterns (11). DQB1*02:01 is linked to DQA1*05:01 and DRB1*03:01, while DQB1*02:02 is typically linked to DQA1*02:01 and DRB1*07:01, each of these combinations constitutes a distinct HLA haplotype. DQ2.5 is referred to the heterodimer formed by the DQB1*02:01 and DQA1*05:01 alleles, while DQ2.2 is for DQB1*02:02 and DQA1*02:01 (11). In this manuscript, we will use clinical abbreviations to designate four haplotypes associated with CeD, including DQ2.5 (DQA1*05:01 and DQB1*02:01), DQ2.2 (DQA1*02:01 and DQB1*02:02), DQ8.1 (DQA1*03:01 and DQB1*03:02), and DQ7.5 (DQA1*05:05 and DQB1*03:01). When an individual carries DQ2.2 on one chromosome and DQ7.5 on the other, a *trans-*encoded DQ2.5 heterodimer can be formed through pairing of the DQA1*05:05 allele from DQ7.5 with the DQB1*02:02 allele from DQ2.2. DQA1*05:05 in DQ7.5 is highly similar to DQA1*05:01 in DQ2.5, allowing the formation of a DQ2.5 heterodimer *in trans*, which confers increased CeD risk compared with DQ2.2 alone (11).

Current clinical guidelines recommend a uniform screening approach for all first-degree relatives, relying on periodic serologic testing (tissue transglutaminase immunoglobulin A [tTG-IgA]) (12, 13). However, this “one-size-fits-all” strategy has significant drawbacks. It subjects family members to increased anxiety and periodic blood draws, even though many may not carry genetic risks and thus face virtually no risk of developing the disease (13). Furthermore, serology-only monitoring does not account for the substantial heterogeneity in risk conferred by specific HLA-DQ combinations (14, 15). These 4 haplotypes, inherited across two chromosomes, can generate 14 distinct haplotype combinations. Our study, together with others, demonstrates that these combinations represent 14 distinct HLA-DQ genotypes, each associated with a different level of risk for CeD, with the highest risk observed in individuals homozygous for DQ2.5 (14, 15, 16). Consequently, a serology-only approach is inefficient for individuals with the highest genetic susceptibility, who may be monitored more frequently to identify early-onset cases (17). Genetic risk stratification may provide a practical way to address these gaps, especially with rapidly decreasing sequencing costs (18). In contrast to traditional allele-specific PCR approaches, Whole-exome sequencing (WES) enables comprehensive assessment of the entire HLA region, facilitates familial segregation and haplotype analysis of both common and rare variants, and allows genetic ancestry inference (19, 20). In this study, we evaluated the clinical utility of WES by enrolling participants from the same families and estimating genetic risk among at-risk first-degree relatives.

## Results

### Demographic and genetic ancestry analysis

**Figure 1.**
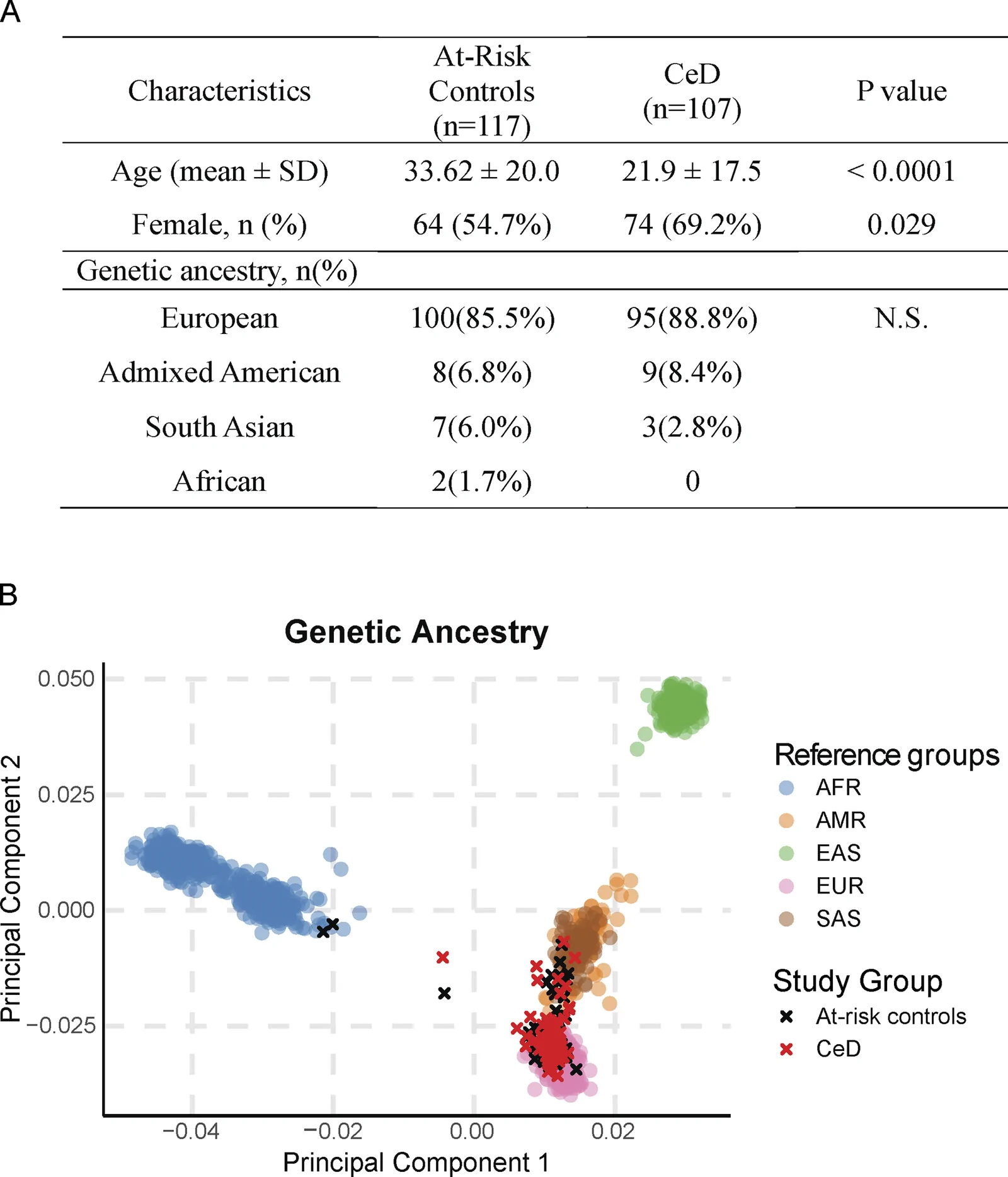
**Characteristics and genetic ancestry of study participants. (A)** Table summarizing the demographic characteristics of the two study groups. **(B)** Genetic ancestry of the participants compared with reference populations from the HapMap Project. AFR, African; AMR, Admixed American; EAS, East Asian; EUR, European; SAS, South Asian; N.S., not significant.

A total of 224 participants were analyzed, including 107 individuals with CeD and 117 at-risk controls. The mean age of CeD cases was 21.9 ± 17.5 years, younger compared with 33.6 ± 20.0 years among controls (P < 0.0001). Among the CeD cases, 33 (30.8%) were male, and 74 (69.2%) were female. In the at-risk control group, 53 individuals (45.3%) were male and 64 (54.7%) were female (P = 0.029). Genetic ancestry analysis showed that 195 (87%) individuals were of European ancestry, 17 (8%) of Admixed American ancestry, 10 (4%) of South Asian ancestry, and 2 (1%) of African ancestry (Fig. 1). In our study, we enrolled all participants who came to our care and were interested in participating in our study, yet we found that 88.9% of CeD cases were of European ancestry, consistent with earlier reports, those of European ancestry having the highest risk (1, 3).

### The differential distribution of HLA-DQ genotypes in CeD cases and at-risk controls

All 107 individuals with CeD carried at least one of the four CeD HLA-DQ risk haplotypes. 71 CeD cases (66%) compared with 48 (41%) at-risk controls, carry at least one HLA-DQ2.5 in *cis* haplotype, while DQ2.5 *in trans* was observed in 11 (10%) CeD cases, including 7 females, and in 3 (2%) controls. HLA-DQ genotypes were grouped into high-, moderate-, and low-risk tiers using a reference-based classification (Table 1 and Table S2). Using this risk-tier framework, 24 CeD cases (22.4%) and 12 at-risk controls (10.3%) carry the high-risk DQ2.5/DQ2.5 or DQ2.5/DQ2.2 genotype. Moderate risk was observed in 69 cases (64.4%) and 42 controls (35.9%), while low-risk DQ genotypes were found in 14 cases (13.1%) and 45 controls (38.5%). In addition, 18 at-risk controls (15.4%) carried none of the four CeD HLA-DQ risk haplotypes. The four-tier risk classification significantly improved predictive accuracy compared to the classical binary approach, achieving an receiver operating characteristic (ROC)-AUC of 0.72 versus 0.58.

**Table 1.** HLA-DQ genotype distribution in CeD cases and at-risk controls

Risk	HLA-DQ genotype	At-risk controls *n* = 117 (*n*)%	CeD *n* = 107 (*n*)%	OR (95% CI)	P value
High	2.5/2.5	4 (3.4%)	8 (7.5%)	6.43 (2.57–16.07)	7.44E-05
	2.5/2.2	8 (6.8%)	16 (15.0%)		
Moderate	2.5/X	22 (18.8%)	32 (29.9%)	5.28 (2.59–10.76)	2.14E-06
	2.2/7.5	3 (2.6%)	11 (10.3%)		
	2.5/7.5	7 (6.0%)	5 (4.7%)		
	2.5/8.1	7 (6.0%)	10 (9.3%)		
	8.1/8.1	1 (<1%)	5 (4.7%)		
	2.2/8.1	2 (1.7%)	6 (5.6%)		
Low	8.1/X	10 (8.5%)	6 (5.6%)	1	Reference
	7.5/8.1	1 (<1%)	2 (1.9%)		
	2.2/X	21 (17.9%)	3 (2.8%)		
	7.5/X	9 (7.7%)	1 (<1%)		
	2.2/2.2	2 (1.7%)	2 (1.9%)		
	7.5/7.5	2 (1.7%)	0 (0%)		
None	X/X	18 (15.4%)	0 (0%)	​	​

*n*, number. Statistical significance was determined by Fisher’s exact test.

### HLA-B8 and AH8.1 long haplotype in CeD

**Figure 2.**
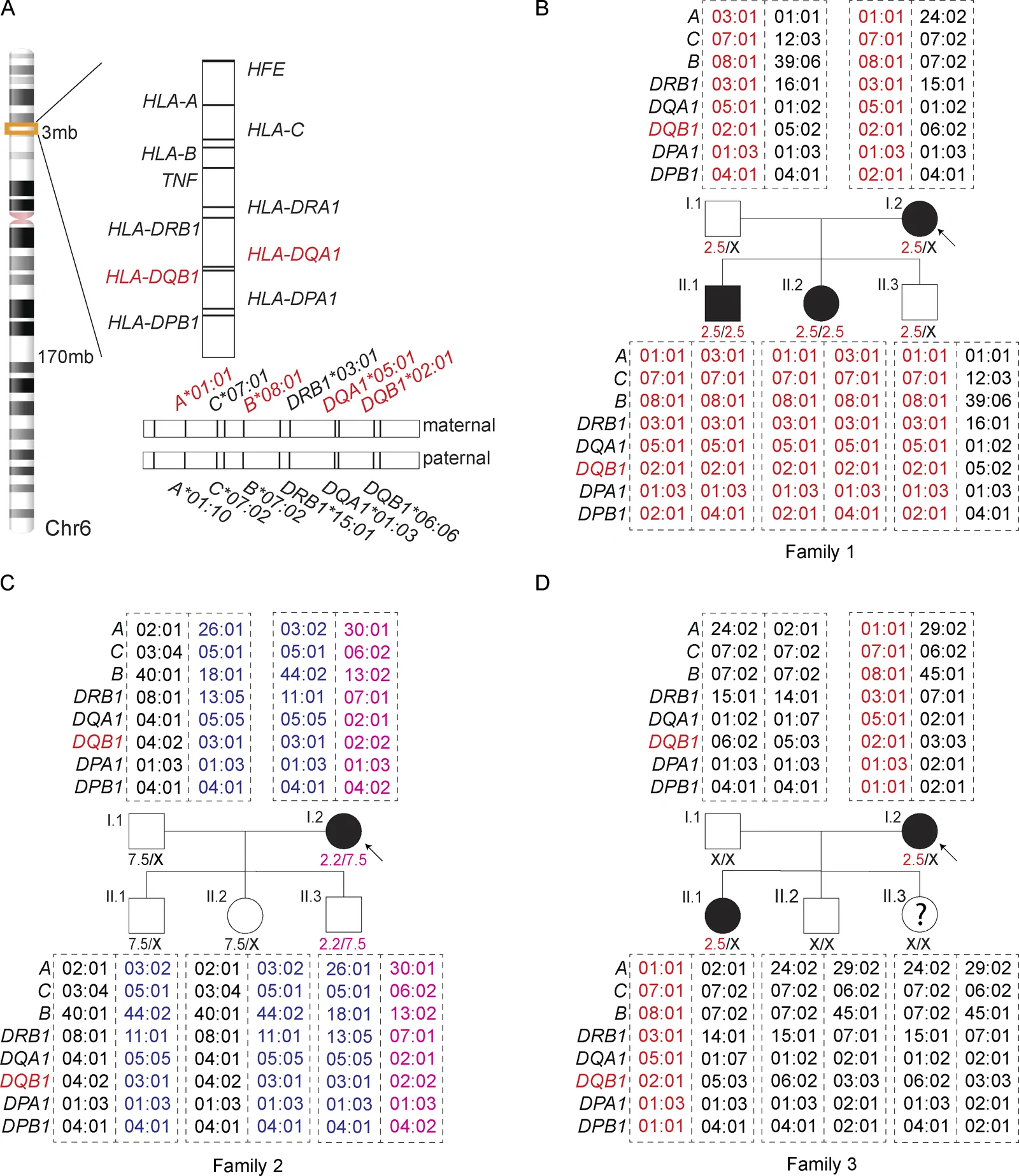
**HLA genes and familial segregation of HLA alleles in three families. (A)** Schematic representation of the HLA gene region on the short arm of chromosome 6. The lower right panel shows the maternal haplotype carrying the extended AH8.1 haplotype with characteristic linkage disequilibrium. **(B)** Family 1, showing two affected individuals carrying the high-risk DQ2.5/2.5 genotype. **(C)** Family 2, in which the index case carries DQ2.5 *in trans* due to the DQ2.2/7.5 combination. **(D)** Family 3, showing a child with abnormal DGP antibodies but without a DQ risk haplotype. Arrows indicate the index cases. DGP, deamidated gliadin.

We found a significant enrichment of HLA-B*08:01 in those with CeD. The allele frequency was 32.2% alleles in the CeD cases, compared with 16.7% in at-risk controls (odds ratio [OR] = 2.38; P = 0.0002, Table 2). This finding is consistent with an earlier study of 12,016 CeD cases and 11,920 controls: HLA-B*08:01 had an allele frequency of 39% in cases versus 12% in controls (21). While analyzing the familial segregation of HLA genes, we identified the extended AH8.1 haplotype A1–B8–C7-DR3–DQ2, which includes HLA-A*01:01 (A1), HLA-C*07:01 (C7), HLA-B*08:01 (B8), HLA-DRB1*03:01 (DR3), HLA-DQA1*05:01, and HLA-DQB1*02:01 (DQ2.5) (22). Among individuals of European ancestry, 62% of the 74 DQB1*02:01 alleles observed in cases carry this extended haplotype. In contrast, 49% of at-risk controls carry the AH8.1 haplotype (Table 3 and Fig. 2 A). Our results from this familial CeD cohort and the All of Us research program, together with prior studies, indicate an additive effect of *HLA-B8* on DQ2.5 within this extended haplotype, thereby increasing genetic risk for CeD (23, *Preprint*, 24).

**Table 2.** Differences in HLA allele frequencies between CeD cases and at-risk controls

HLA allele	At risk controls % (*n* = 117)	CeD % (*n* = 107)	OR (95% CI)	P value
B*08:01	16.67%	32.24%	2.38 (1.52–3.72)	0.0002
DRB1*03:01	22.65%	36.92%	1.99 (1.32–3.02)	0.0010
A*01:01	19.66%	31.78%	1.91 (1.24–2.94)	0.0034
DQB1*02:01	22.22%	36.45%	2.01 (1.33–3.04)	0.0012
C*07:01	20.09%	30.84%	1.77 (1.15–2.73)	0.0092
DQB1*02:02	16.81%	18.87%	1.15 (0.71–1.87)	0.6199
DQA1*05:01	23.08%	36.45%	1.91 (1.27–2.89)	0.0026

Statistical significance was determined by Fisher’s exact test.

**Table 3.** The AH8.1 long haplotype in European participants

*DQB1* 02:01 alleles^a^	At-risk controls (*n*)% *n* = 49	CeD (*n*)% *n* = 74
DRB1*03:01	49 (100%)	73 (98.6%)
HLA-B*08:01	34 (69%)	59 (80%)
HLA-C*07:01	36 (74%)	54 (73%)
HLA-A*01:01	24 (49%)	50 (68%)
ALL Present	24 (49%)	46 (62%)

a The total number of *DQB1* 02:01 alleles observed.

### A family-based interpretation for CeD genetic risks

To further illustrate the differential risks among first-degree relatives, we selected three representative families. In family 1, the index case (I.2, Fig. 2 B) carried a DQ2.5/X genotype; her husband also carries one copy of DQ2.5. Their children (II.1 and II.2) inherited one copy of DQ2.5 from each parent, have the high-risk homozygous DQ2.5/2.5 genotype, and have already developed CeD. While II.3 carries one copy of DQ2.5/X, a moderate risk for CeD, supporting periodic screening. In family 2, the index case (I.2, Fig. 2 C) carried DQ2.2/7.5; DQB1*02:02 and DQA1*05:05 encode DQ2.5 *in trans* making her susceptible to CeD. Among her three children, II.3 carried DQ2.2/7.5, forming DQ2.5 *in trans*, placing the child at moderate-risk. The other two children (II.1 and II.2) carry the DQ7.5/X genotype, which is considered low-risk for developing CeD. In family 3, a child (II.3, Fig. 2 D) was diagnosed with CeD based on an elevated deamidated gliadin peptide antibody level and a strong family history, despite normal tTG-IgA levels. The patient does not carry any of the risk haplotypes. The patient does not have CeD, and genetic testing would prevent an incorrect diagnosis (12).

### WES-based polygenic risk score

**Figure 3.**
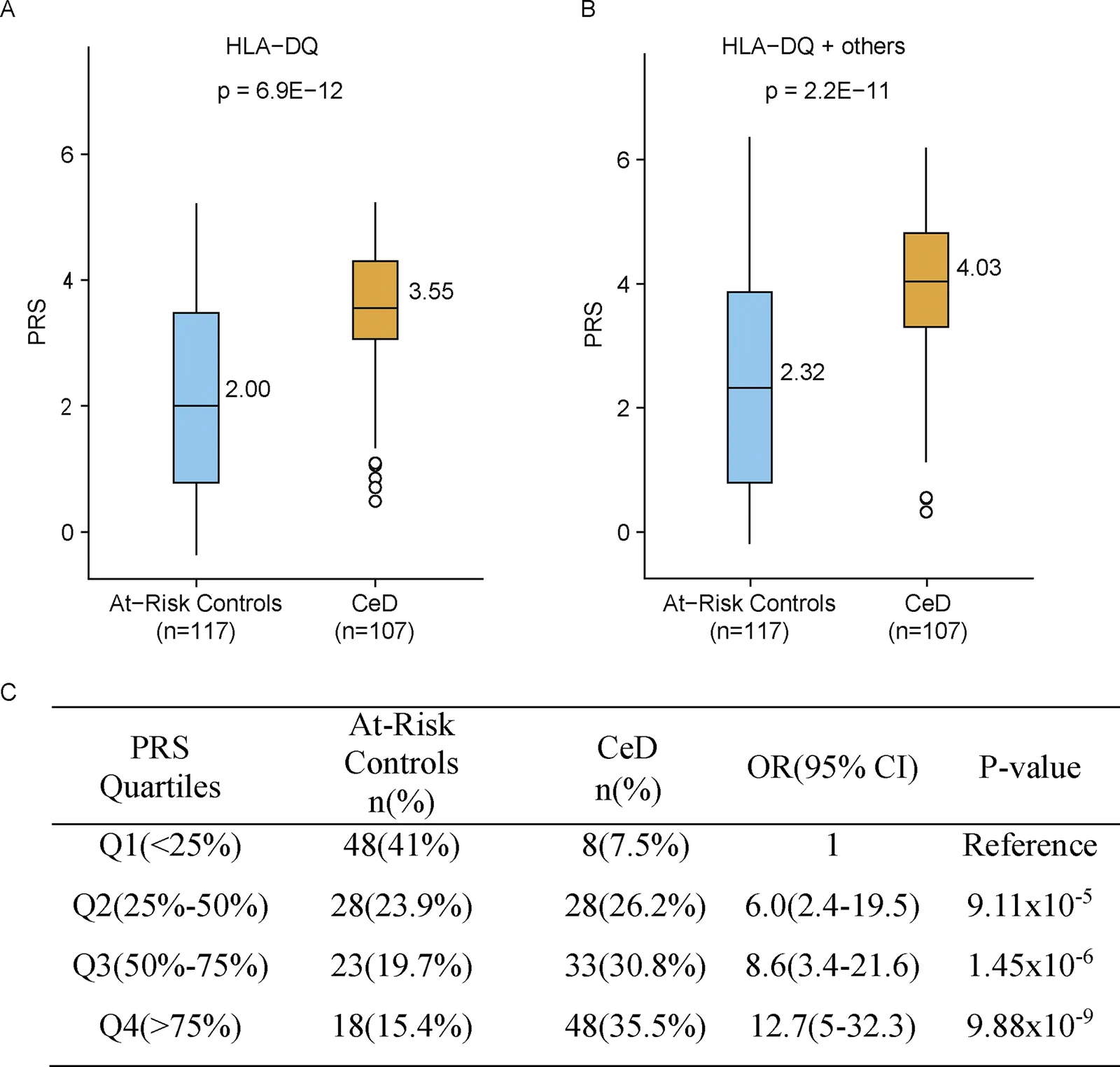
**Genetic risk scoring for CeD risk stratification. (A)** PRS based on 14 HLA-DQ genotypes. **(B)** PRS based on 14 HLA-DQ genotypes, HLA-B*08:01, and five additional non-HLA SNPs, difference between CeD cases and at-risk controls were evaluated using Student’s *t* test to compare mean values. **(C)** Distribution of PRS quartiles in the CeD and control groups.

To better assess the cumulative genetic risk, we evaluated a polygenic risk score (PRS) model based on 14 HLA-DQ genotypes and 38 associated single nucleotide polymorphism (SNPs) with optimal predictive value in European individuals (14). While HLA-DQ genotypes can be called by WES, only four of 38 associated SNPs were covered due to most of these SNPs being in introns. We then searched for exonic variants in linkage disequilibrium with those intronic SNPs; only one additional SNP was found (r^2^ = 0.90). Furthermore, HLA-B*08:01 status was determined via HLA*LA typing. We evaluated a modified scoring model that included HLA-B*08:01 and five SNPs in addition to the HLA-DQ genotypes. The addition of these variants did not significantly enhance predictive performance, confirming that the model is primarily driven by HLA-DQ (14). Both analyses revealed a significantly higher median PRS in CeD cases compared with at-risk controls (Fig. 3, A and B). Compared with individuals in the lowest-risk quartile (Q1, <25%), those in higher quartiles had significantly increased odds of CeD: 6.1-fold higher in Q2 and 8.6-fold higher in Q3. The greatest risk was observed in the highest quartile (Q4), where individuals had a 12.7-fold increase in odds of CeD (Fig. 3 C). These results suggest that genetic risks for CeD can be better evaluated by considering the differential impact of risk genotypes.

## Discussion

Our analysis demonstrates that WES effectively resolves HLA typing and characterizes HLA linkage disequilibrium within families, revealing the effect of HLA-B8. At the same time, WES enables genetic ancestry inference and provides more precise risk stratification than assessment based solely on HLA-DQ2/8 (19). HLA-DQ genotypes have been shown to predict the risk of CeD. The longitudinal TEDDY study followed 6,403 children from four countries with four HLA genotypes: DR3/4, DR4/4, DR4/8, and DR3/3. Based on the known linkage disequilibrium structure of the HLA region, the DR3/4 genotype is equivalent to DQ2.5/8.1, while DR4/4 corresponds to DQ8.1/8.1 and DR3/3 corresponds to DQ2.5/2.5. By age 5 years, the cumulative risk of developing positive tTG-IgA serology was 26% among children homozygous for DQ2.5 and 11% among those with DQ2.5/8.1. These findings support a differential gene dosage effect across distinct HLA-DQ genotypes (24). The PreventCD birth cohort studied 944 genetically predisposed children from affected first-degree relatives. 35.4% with DQ2.5/DQ2.5 or DQ2.5/2.2 genotype developed CeD. A personalized prediction of CeD was developed based on sex, the number of affected first-degree relatives, and five HLA risk groups (17). The preventCD study recommends that first-degree relatives with the highest-risk DQ genotype be screened for tTG-IgA every 6 mo during childhood, while those with a low-risk genotype be screened every 2 years (17). Currently, no next-generation sequencing–based HLA haplotyping data are available from either the PREVENT-CD or TEDDY cohorts. Future investigations should incorporate advanced sequencing technologies for more precise HLA characterization and improved genetic risk stratification in CeD.

Genome-wide association studies involving >100,000 patients with CeD have identified >40 risk loci (25). While the biological impact of any single SNP is limited, their collective contribution provides valuable predictive power. Our data, along with others, support the prediction can be further improved by treating each *HLA-DQ* genotype as an independent risk factor and incorporating additional genetic risks. Romanos et al. classified *HLA-DQ* genotypes into three risk groups (high, intermediate, and none) and incorporated 57 non-HLA SNPs, achieving an area under the AUC of 0.86 in CeD prediction (26). Abraham et al. constructed a PRS using ∼200 SNPs, including HLA tagSNPs instead of HLA-DQ genotypes, which modestly improved performance to an AUC of 0.87 (27). Sharp et al. further advanced this approach by integrating 14 HLA-DQ genotypes (based on four tag SNPs), five non-DQ HLA variants, and 33 SNPs outside the HLA region, achieving an AUC of 0.88 for distinguishing CeD cases from controls (14). The recent HUNT study reported no significant difference in PRS based on 1,582 SNPs when comparing previously diagnosed CeD cases with newly diagnosed cases, with the model achieving an AUC of 0.86 (28). From our analysis of All of Us data, we modified Sharp’s model by computing 14 HLA-DQ genotypes using HIBAG, achieving an AUC of 0.86 for discriminating seropositive CeD patients from non-CeD controls (23, *Preprint*). While WES provides coverage of HLA-DQ genotypes and HLA class I and II loci, it does not cover the majority of intronic SNPs used for PRS calculations; moreover, no coding variants in LD with these associated SNPs could be identified.

The optimal interval for tTG-IgA screening should be further studied. Sequencing of DNA obtained from saliva represents a low-risk, noninvasive approach for predicting CeD risk. With the FDA approval of GlutenID (29) and the declining cost of genetic sequencing, an increasing number of individuals will have access to HLA-DQ genotyping or CeD genetic risk scores. This provides an opportunity to longitudinally follow individuals with different HLA-DQ genotypes and to better determine the optimal interval and the risk–benefit of periodic screening. Furthermore, future studies should include a higher number of non-European celiac patients, as this study included a very small number of non-European individuals.

In the future, additional approaches may further improve CeD risk prediction, including comprehensive *in vitro* immunophenotyping, assessment of gluten-specific memory T cell responses, and integration of RNA-seq data to functionally interpret both common and rare CeD risk loci. Future clinical trials can evaluate the potential benefits of initiating a preemptive gluten-free diet in high-risk individuals compared to standard implementation following formal diagnosis. Ultimately, these complementary strategies may help advance a model of precision prevention, enabling more efficient allocation of healthcare resources, reducing unnecessary anxiety, and improving the diagnostic accuracy of CeD for clinical management.

## Materials and methods

### Study design and cohort

This study was approved by the UT Southwestern Medical Center Institutional Review Board (ID: STU112010-130). Written informed consent was obtained from all participants enrolled in the study. CeD cases were defined based on a prior clinical diagnosis supported by serologic testing and/or histologic confirmation. Controls were first-degree relatives of CeD cases. A total of 227 individuals were enrolled from 2022 to 2025. One individual was excluded due to insufficient sequencing coverage. Further review of two cases diagnosed with CeD in the absence of HLA-DQ risk variants revealed a lack of characteristic serologic and pathological features. These individuals were excluded from the final analysis. The final analytic cohort, therefore, included 224 individuals. The cohort was predominantly family-based, with 209 individuals belonging to 66 families.

### Genetic sequencing and genetic ancestry analysis

Genomic DNA was extracted from saliva samples collected using an Oragene collection kit (ON-500, Genoteck). Genomic DNA samples were sent to Azenta or Novogene for DNA fragmentation, library preparation, and capture-based WES on the Illumina platform. Raw read quality was assessed using FastQC and MultiQC (30, 31). Data were processed using a GATK-based pipeline implemented on a high-performance computing cluster. Following base quality score recalibration, variants were called using HaplotypeCaller and jointly genotyped using GenotypeGVCFs, with exome target regions restricted to 100 bp padding. The resulting variant call format (VCFs) were normalized, and multiallelic variants were split using bcftools (32). The final output consisted of high-quality, per-sample VCFs prepared for downstream analysis. Data from sequencing batches using different capture kits were harmonized and merged, with one batch serving as the reference. Population structure was assessed via principal component analysis using the HapMap reference panel, following the reported protocols (33, 34). Data management and merging were performed using PLINK v1.9 (35).

### HLA-DQ genotyping

High-resolution HLA class I and class II genotypes were inferred from WES data using the graph-based algorithm HLA*LA (36). We assessed sequencing quality by calculating read depth across all target loci. The overall average coverage was ∼71.8× for class I genes. The average coverage across all class II genes was slightly lower (∼49.7×), with highly variable loci such as *DQA1* and *DQB1* averaging 31.9× and 48.7×, respectively, due to known exome-capture biases in the highly polymorphic HLA region, yet it remained sufficient for reliable allele calling (37). Detailed coverage statistics per gene are provided in Table S1.

### Statistics

Statistical analyses were performed using R software (version 4.5.1). Continuous variables are presented as means ± standard deviation (SD) or medians, while categorical variables are expressed as counts and percentages. Differences in genotype distributions between cases and controls were evaluated using Fisher’s exact test. To quantify the strength of associations, ORs and 95% confidence intervals were calculated. To generate a comprehensive reference dataset for HLA-DQ genotype risk evaluation, we aggregated case-control data from two published reports (14, 15), yielding a total of 16,540 CeD cases and 22,673 controls enrolled from the United Kingdom, Finland, the Netherlands, and Italy (Table S2). By calculating ORs for the 14 HLA-DQ genotypes, we stratified the genotypes into a four-tier classification based on effect size: High-risk (OR >90), moderate-risk (OR 9–36), low-risk (OR 1–5), and no risk (OR <1). For the ROC analysis, four tiers were assigned ordinal risk weights of 3, 2, 1, and 0, respectively. For comparison with standard binary classification, we assigned a binary risk weight of 1 for the presence and 0 for the absence of any of the four CeD-associated haplotypes (HLA-DQ2.5, DQ2.2, DQ8, or DQ7.5).

### Online Supplemental material

Table S1 details the sequencing coverage statistics. Table S2 presents the detailed HLA-DQ genotype frequencies and ORs from two previous studies. Table S3 provides annotation information about five of the 38 CeD-associated SNPs.

## Supplementary Material

Table S1 details the sequencing coverage statistics.

Table S2 presents the detailed HLA-DQ genotype frequencies and ORs from two previous studies.

Table S3 provides annotation information about five of the 38 CeD-associated SNPs.

## Data Availability

All authors disclose no conflicts of interest. The data generated during the current study are not publicly available due to ongoing analysis.
